# Exploration of the impact of political ideology disparity on COVID-19 transmission in the United States

**DOI:** 10.1186/s12889-022-14545-3

**Published:** 2022-11-24

**Authors:** Rongxiang Rui, Maozai Tian, Wei Xiong

**Affiliations:** 1 School of Statistics, Renmin University of China, No. 59 Zhongguancun Street, Haidian District, Beijing, 100872 P.R. China; 2 Department of Medical Engineering and Technology, Xinjiang Medical University, Urumqi, 830011 China; 3 School of Statistics, University of International Business and Economics, Beijing, China

**Keywords:** COVID-19 Crisis, Disparity of Political Ideology, Functional Data Analysis, Post-Omicron Period, Seasonal Periodicity, Unit Infection Rate

## Abstract

**Background:**

Based on individual-level studies, previous literature suggested that conservatives and liberals in the United States had different perceptions and behaviors when facing the COVID-19 threat. From a state-level perspective, this study further explored the impact of personal political ideology disparity on COVID-19 transmission before and after the emergence of Omicron.

**Methods:**

A new index was established, which depended on the daily cumulative number of confirmed cases in each state and the corresponding population size. Then, by using the 2020 United States presidential election results, the values of the built index were further divided into two groups concerning the political party affiliation of the winner in each state. In addition, each group was further separated into two parts, corresponding to the time before and after Omicron predominated. Three methods, i.e., functional principal component analysis, functional analysis of variance, and function-on-scalar linear regression, were implemented to statistically analyze and quantify the impact.

**Results:**

Findings reveal that the disparity of personal political ideology has caused a significant discrepancy in the COVID-19 crisis in the United States. Specifically, the findings show that at the very early stage before the emergence of Omicron, Democratic-leaning states suffered from a much greater severity of the COVID-19 threat but, after July 2020, the severity of COVID-19 transmission in Republican-leaning states was much higher than that in Democratic-leaning states. Situations were reversed when the Omicron predominated. Most of the time, states with Democrat preferences were more vulnerable to the threat of COVID-19 than those with Republican preferences, even though the differences decreased over time.

**Conclusions:**

The individual-level disparity of political ideology has impacted the nationwide COVID-19 transmission and such findings are meaningful for the government and policymakers when taking action against the COVID-19 crisis in the United States.

## Background

Since the end of 2019, COVID-19 has become the most enormous pandemic in the world and the severity of the COVID-19 crisis is still not diminished so far. Such a global crisis corresponds to, as of October 6, 2022, more than 6 million deaths and over 600 million infections [1]. According to information about the COVID-19 from World Health Organization [1], one can see that the first three countries with the highest number of confirmed cases are the United States (US), India, and France. Particularly, over 15% of cumulative total cases around the world are coming from the US, which has caught the eyes of many researchers to find out the possible factors that could be significantly associated with COVID-19 transmission in the US.

Even though the first vaccine produced in the United States was available early in December 2020 and the vaccination coverage rate and vaccination intention had increased [2], the infection rate has not decreased significantly. This contradiction could be due to the small number of people who were completely vaccinated in the early stage. However, more and more literature suggests that ideological discrepancy, among others, was also a key factor affecting perceptions of the COVID-19 threat and responses to government orders and guidelines [3, 4], which could further affect the spread of COVID-19 and lead to more infections.

In this respect, some studies analyzed the perception differences of the COVID-19 pandemic in different population groups with various political ideologies. Painter and Qiu [5] found that Democrats tended to have less compliance with the state-level order when it was issued by a Republican governor relative to one from a Democratic governor. Grossman et al. [6] revealed that the reduction in mobility was much more significant in Democratic- than Republican-leaning counties. Latkin et al. [7] found that politically conservative people were more likely to believe skeptical information about COVID-19 and less likely to implement prevention actions to mitigate the possibility of the COVID-19 threat. Besides, pseudo-scientific statements, conspiracy theories, risk perceptions, and skepticism of vaccination policies were also much more prevalent among political conservatives. Gao and Radford [8] discovered that higher death rates were shown in counties with higher levels of Trump support. Fridman et al. [9] found that Republicans showed a negative attitude toward vaccines as the increased salience of the COVID-19 threat. Cai et al. [10] revealed that conservative ideology and political behavioral commitment could jointly lead to less compliance with social distance orders in weaker community-logic communities. Painter and Qiu [11] found that citizens in Republican-leaning counties were less likely to completely obey the stay-at-home order relative to those in Democratic-leaning ones. Kerr et al. [12] revealed that liberals had higher risk awareness, less trust in politicians to address COVID-19, more trust in medical experts, and more criticism of the government response. Kiviniemi et al. [13] found that the more one perceived oneself as a Republican, the less one recognized the risk to oneself and others from COVID-19.

The aforementioned studies mainly focus on revealing the phenomenon of the presence of different attitudes to the COVID-19 crisis due to the disparity of ideology. However, clarifying the differences in outcomes due to the disparity of ideology is also no doubt important. It can not only justify the conclusions of many individual-level studies but also systematically describe the differences in the severity of the COVID-19 crisis in different regions. Furthermore, it also can give administrations evidence, in terms of the overall picture, that all people have to pay close attention to such disparity when trying to contain the spread of COVID-19 in the US by implementing suitable strategies.

Nevertheless, outcomes corresponding to such differences are rarely considered. Some reasons could be no suitable methodology or the dearth of correlated data and indices. This present study focused on this issue and used methodologies correlated to functional data analysis to find out the outcomes of such ideological disparity. The data considered is about the state-level cumulative confirmed cases derived from the surveillance administrative Centers for Disease Control and Prevention [14]. Together with a demographic dataset, a new index was established to reflect the severity of the COVID-19 pandemic, which is inspired by Neelon et al. [15]. The data was divided into two parts to perform analysis respectively according to the day, i.e. November 26, 2020, when WHO [16] announced the variant of SARS-CoV-2 named Omicron became a variant of concern. Furthermore, political beliefs following the 2020 US presidential election results were considered to divide the data into two parts corresponding to Republican- and Democratic-leaning states respectively. Based on the constructed index, this paper separately analyzed the differences in COVID-19 transmission before and after the emergence of Omicron due to ideological discrepancies and further analyzed whether such differences followed the same pattern before and after the emergence of Omicron.

## Materials and methods

### Data description

Three datasets were utilized. The first one was the 2020 US presidential election data, which in detail recorded the votes of each presidential candidate corresponding to each state and can be downloaded from the MIT Election Lab [17]. The second was the state-level population data for the year 2020, which can be downloaded from the United States Census Bureau [18]. Since the actual population size of each state was unavailable, the corresponding estimate from the USCB was applied. The third one was the daily cumulative confirmed cases from all over the US states, which can be openly downloaded from the CDC [14]. Because, in very early 2020, the COVID-19 pandemic was not widely spread in the US and the date that President Donald Trump declared a state emergency was on March 13, the surveillance data of state-level daily cumulative confirmed cases from 21 March 2020 to 6 October 2022, were used in this research.

It should be noted that the emergence of Omicron in 2021, which is a new variant of SARS-CoV-2, has caused an entirely different situation for the risk of infection as well as death. Directly analyzing the data in the whole timespan could be unreliable. Therefore, the considered data is further partitioned into two parts following the day, i.e. 26 November 2021, when WHO [16] announced that Omicron had become a variant that showed significant differences compared to SARS-CoV-2 and other relevant variants. Furthermore, the total cumulative confirmed cases for each state on November 26, 2021, are subtracted from the data corresponding to the time when Omicron predominated. Thus, the data corresponding to the post-Omicron period is only about the pure cumulative confirmed cases after that day.

**Fig. 1:**
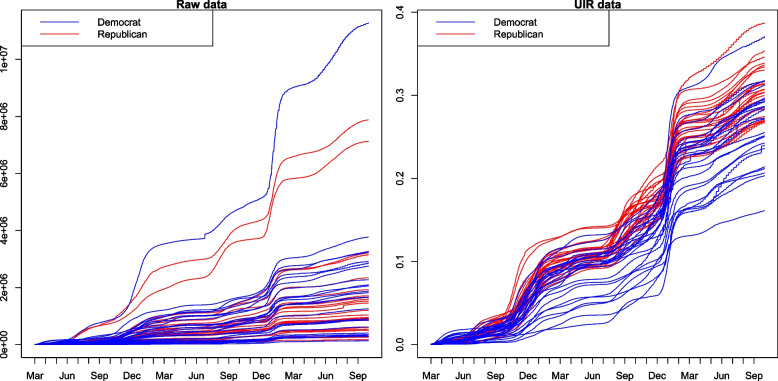
The raw and UIR data of 50 states corresponding to Democratic- and Republican-leaning states

Since the scale of the raw data of the state-level daily cumulative confirmed cases depends on the population size of each corresponding state, i.e. states with a larger population tend to have more confirmed cases, it is almost impossible to find any informative results by using the raw data (see in the left panel of Fig. 1). Therefore, inspired by Neelon et al. [15], an index called unit infection rate (UIR) that mitigates the impact of population size was constructed:

1 $$UIR=(\textit{the state-level daily cumulative confirmed cases})/(\textit{the population of the related state})$$

**Fig. 2:**
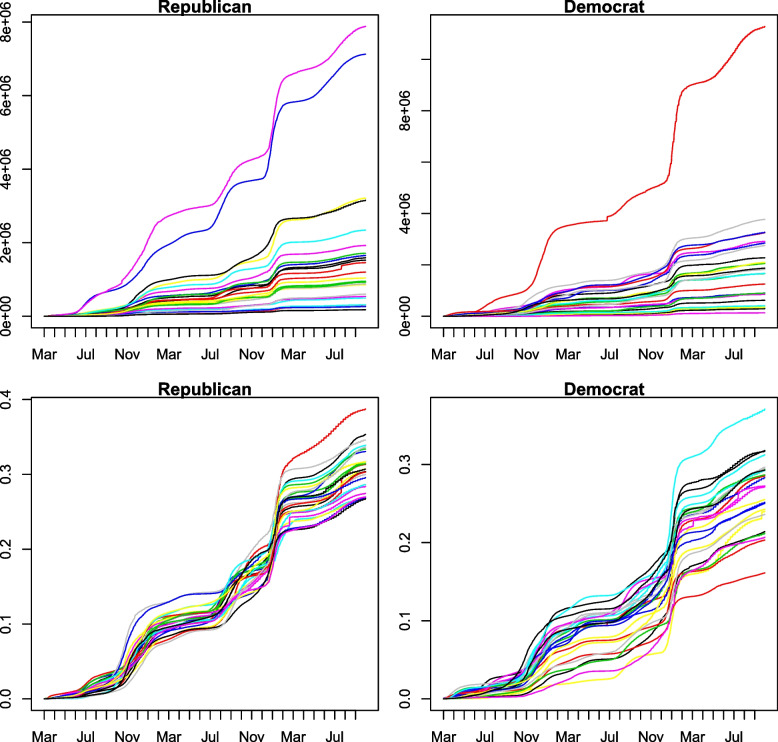
The raw (top) and weighted (bottom) state-level daily cumulative data corresponding to Democratic- and Republican-leaning states

The right panel of Fig. 1 shows UIR curves for Republican- and Democratic-leaning states. One can see that the modified data show strong evidence of differences across states. Particularly, lines for Republican-leaning states are mostly over that for Democratic-leaning ones, which implies the possible existence of the heterogeneity of the COVID-19 severity between Republican- and Democratic-leaning states. Figure 2 exhibits the differences between before and after weighting for each state.

### Methodology

Three steps were considered to analyze the difference of COVID-19 spread in different states corresponding to Democratic- and Republican-leaning states respectively. The first step used the functional principal component analysis [19] method to separately explore the transmission pattern in Republican- and Democratic-leaning states. Then, one can roughly see whether the differences exist between these two sides.

The second step applied the functional analysis of variance (ANOVA) method to identify the significance of the diversity between Democrat- and Republican-leaning regions. Concretely, various testing methods were considered to test the existence of significant differences between Democratic- and Republican-leaning states, which include the L2 norm-based test (L2b) [20], the bootstrap F-type test (Fb) [20] the permutation test (FP) [21] the globalizing pointwise F test (GPF) [22] the naive F-type test (FN) [23] and the bias-reduced F-type test (FB) [24].

The third step implemented a functional linear regression model [25] to quantify the differences between Republican- and Democratic-leaning states. To do this, a nominal predictor (NP) indicating the political party affiliation was engaged. That is,

2 $$UIR(time)= Intercept(time)+Slope(time) \times NP + Error(time),$$

where the NP is equal to 1 if a state is Republican-leaning and 0 if the state is Democratic-leaning.

Some pre-assumptions for UIR are proposed here. First, the observed data is assumed to be functional, i.e., each observation of a state is a smooth function over time, which guarantees the availability of further analysis using approaches for functional data. Second, each observed UIR in the same group is little correlated. Such an assumption can be derived from the previous study by Rui et al. [26] whose findings revealed that the number of confirmed cases for a single state almost relied on the number of previous cases of that state itself.

## Results

### Disparity seen from functional PCA

**Fig. 3:**
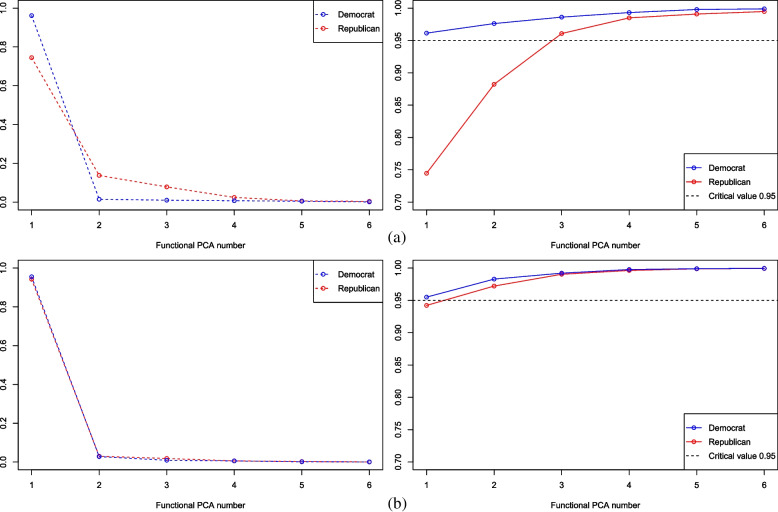
Variance explanation for Democratic- and Republican-leaning states before (**a**) and after (**b**) Omicron predominated

To show the descriptive statistics of the severity of the COVID-19 pandemic in Democratic- and Republican-leaning regions based on functional PCA, one first has to determine the number of functional principal components. For the time before the emergence of Omicron, according to the proportion of variance explained by each eigenfunction shown in the left panel of Fig. 3(a), the proportion of the first eigenfunction is 0.961 for Democratic-leaning states (0.745 for Republican-leaning states), that of the second eigenfunction is 0.015 for Democratic-leaning states (0.138 for Republican-leaning states), and that of the third eigenfunction is 0.010 for Democratic-leaning states (0.079 for Republican-leaning states). In this regard, the proportion of the explained variance about the leading three eigenfunctions for Democratic- and Republican-leaning states is 0.986 and 0.961 separately (see the right panel of Fig. 3(a)). Furthermore, one can see that the tendency of variance explanation of each eigenfunction in both regions suddenly slows down after the first three eigenfunctions. It follows that considering the first three eigenfunctions is enough. Similarly, the number of eigenfunctions corresponding to the post-Omicron period can be fixed as 2 based on their proportion of explained variance shown in Fig. 3(b).

**Fig. 4:**
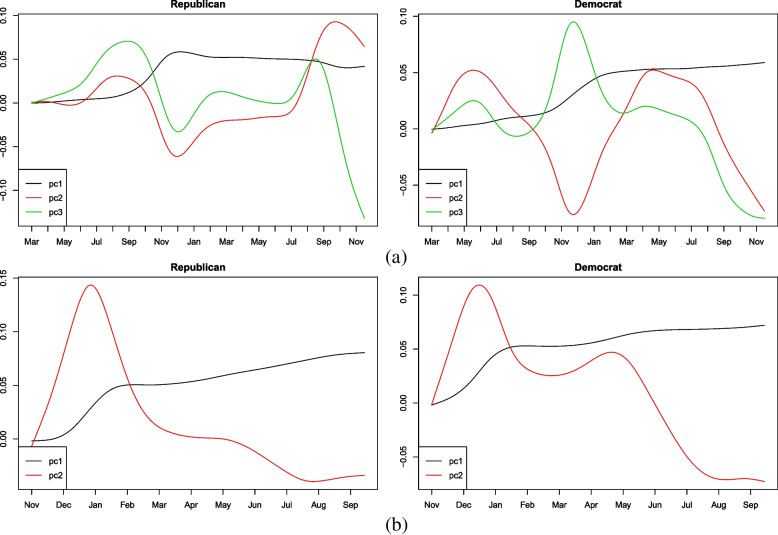
Selected eigenfunctions with respect to different party preferences before (**a**) and after (**b**) Omicron predominated

The selected three eigenfunctions for Democratic- and Republican-leaning states before the emergence of Omicron are shown respectively in Fig. 4(a). On the one hand, some common phenomena in both groups are unearthed. For instance, the first eigenfunction shows a rapid increase in the first half, the third eigenfunction exhibits a periodic change, and both the second and the third eigenfunctions present a downward trend near November 2021. For the post-Omicron period, as shown in Fig. 4(b), the first eigenfunction shows an upward tendency before February 2022 and then slows down and the second eigenfunction presents a consistently upward and then downward trend.

On the other hand, even though there are no apparent differences in the shape of eigenfunctions corresponding to the post-Omicron period, the correlated pattern is different for the time before the emergence of Omicron when considering the specific fluctuation of each component pair. For example, the first eigenfunction of Republican-leaning states has the largest growth from around September to December 2020 whereas the period is in around January 2021 for Democratic-leaning states. The pattern of periodic change for the second eigenfunction is also different. There is a plateau from March to June in each year for Republican-leaning states whereas such a scenario is not shown in Democratic-leaning states. Such discrepancies could imply that the differences in political ideology at an individual level have a nationwide impact on COVID-19 transmission in the US.

### Disparity confirmed from functional ANOVA

The previous subsection illustrates some differences between Democratic- and Republican-leaning states. In this part, such discrepancies will be further investigated by using the functional ANOVA method. The null hypothesis for functional ANOVA is that the disparity of UIR is not overall significant between the Democratic- and Republican-leaning states. Thus, if the null hypothesis is not rejected, then the phenomena seen in the previous subsection are not statistically significant on the whole, which implies that political ideology disparity is only a personal-level issue and hasn’t caused the difference of COVID-19 transmission among states with different party preferences. To be specific, the hypothesis testing problem is: *The mean function of the Democratic-leaning states is equal to the mean function of the Republican-leaning states*.

Tables 1 and 2 report the testing results based on the 6 selected methods. According to the *P*-value corresponding to each method, one can see that, with the threshold of 0.05, the differences in COVID-19 transmission between Democratic- and Republican-leaning states are statistically significant during the time before the emergence of Omicron whereas the null hypothesis for the post-Omicron period is statistically unrejected. In short, these consequences show that the disparity of American political ideology influenced the spread of COVID-19 before Omicron predominated and made the COVID-19 threat different between Democratic- and Republican-leaning states whereas the difference of COVID-19 crisis for two sides during the post-Omicron period is not overall significant.

**Table 1:** Functional ANOVA outcomes based on 6 test methods before Omicron predominated

Method	L2b	Fb	FP
*P*-value	0.000	0.000	0.000
Outcome	Rejected^a^	Rejected^a^	Rejected^a^
Method	GPF	FN	FB
*P*-value	1.645e-7	3.665e-6	3.260e-6
Outcome	Rejected^a^	Rejected^a^	Rejected^a^

^a^ The threshold is fixed as 0.05

**Table 2:** Functional ANOVA outcomes based on 6 test methods during the post-Omicron period

Method	L2b	Fb	FP
*P*-value	0.291	0.307	0.291
Outcome	Unrejected^a^	Unrejected^a^	Unrejected^a^
Method	GPF	FN	FB
*P*-value	0.174	0.304	0.304
Outcome	Unrejected^a^	Unrejected^a^	Unrejected^a^

^a^ The threshold is fixed as 0.05

### Disparity quantified from functional LRM

**Fig. 5:**
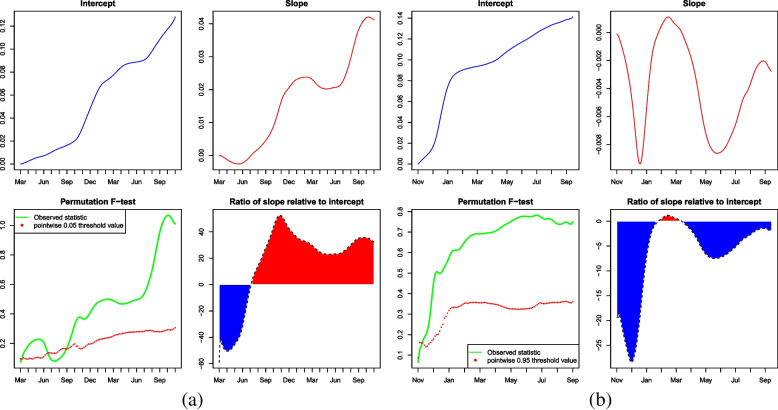
Outcomes based on Functional LRM before (**a**) and after (**b**) the emergence of Omicron

The difference of the COVID-19 crisis between the Democratic- and the Republican-leaning states is quantified here, which is shown in Fig. 5. Since the mean of the error term is assumed to be zero, the intercept function given the value of NP as zero is the mean function of UIR for Democratic-leaning states. One can see that the UIR for Democratic-leaning states rises rapidly during the time before the emergence of Omicron (see the top-left plot of Fig. 5(a)) and such a tendency seems to continue for a long period. Note that most of the time, the permutation F-test shown in the bottom-left plot of Fig. 5(a) indicates the slope function is significantly different from zero, which means that there is a significant difference of UIR between Democratic- and Republican-leaning states. More importantly, under the same settings, the slope function can be considered as the relative difference between Democratic- and Republican-leaning states. It follows that about before July 2020, the UIR for the Democratic-leaning states was higher than that for the Republican-leaning states. However, since then the UIR for Republican-leaning states became higher than that for the Democratic-leaning states.

The bottom panel of Fig. 5(a) is about the pointwise ratio of slope over intercept, i.e. slope/intercept × 100%. Since the intercept is about the overall mean of the Democratic-leaning states and the slope is the increment of the Republican-leaning states relative to the Democratic-leaning ones, the ratio also indicates the proportion of UIR that the Republican-leaning states are more than the Democratic-leaning states, i.e.

3 $$slope/intercept\times 100\% =(\textit{UIR of Republican-leaning states} - \textit{UIR of Democratic-leaning states})/ (\textit{UIR of Democratic-leaning states})\times 100\%$$

In this respect, before July 2020, the ratio was negative. Especially around March 2020, the ratio was smaller than -50%, which implies the situation of COVID-19 transmission in Democratic-leaning states was much severer than that in Republican-leaning states at that moment. However, after that, the ratio dramatically rose to more than 40% and fluctuated up and down, and showed a slow downward trend. However, the UIR for Republican-leaning states was still more than 20% higher than that in the Democratic-leaning ones.

For the scenario during the post-Omicron period, one can see from Fig. 5(b) that the UIR of the Democratic-leaning states showed an upward trend, which was consistent with the situation before Omicron predominated. The pointwise permutation F-test for the slope function was significantly different from zero. However, different from the results before the emergence of Omicron, the slope function didn’t have an obviously increasing or decreasing tendency. In contrast, it showed a sine-like shape and the value was mostly negative in the whole timespan. Accordingly, the ratio indicated the severity of COVID-19 in Democratic-leaning states was almost always higher than that in Republican-leaning states and the degree of this difference tended to decrease over time.

## Discussion

Booming studies pointed out the existence that political discrepancy caused different attitudes when facing the COVID-19 threat. For instance, a study by Grossman et al. [6] showed that political partisanship influenced the degree of compliance with stay-at-home orders and social distancing recommendations. Conway Iii et al. [27] gave evidence that political reasons played a key role in mediating the conservatives’ lack of concern for COVID-19. Nowlan and Zane [28] found that conservatives perceived a greater risk than liberals when facing the COVID-19 threat. Kiviniemi et al. [13] found that political ideology preference was consistently associated with one’s perception of risk from SARS-CoV-2 infection and COVID-19 disease. Piltch-Loeb et al. [29] revealed that political affiliation was one of the most salient factors that relate to decision-making and willingness to vaccination. Albrecht [30] further found that Republican-leaning counties corresponded to significantly lower vaccination rates and much higher cases and deaths than Democratic-leaning states. Kemmelmeier and Jami [31] discovered that conservatives generally corresponded to lower mask-wearing and presented consistently unfavorable attitudes toward mask-wearing. Young et al. [32] further found evidence that Republicans and Trump supporters were less likely to wear a mask. A similar phenomenon was also introduced by Kahane [33] who gave a piece of empirical evidence that in counties with strong support for Trump wearing a mask while in public was remarkably lower.

The purpose of this study is to determine whether the difference of individual political ideology has led to a significant difference in the severity of COVID-19 transmission among states with different party preferences. Findings reveal that before the emergence of Omicron, the severity of COVID-19 transmission in Republican-leaning states is generally higher than that in Democratic-leaning states. This is somewhat consistent with flourishing literature such as studies mentioned above that suggested individual differences in ideology led to different behaviors in the face of the threat of COVID-19. Taken together, one can conclude that before the emergence of Omicron, individual-level ideological differences not only affect a person's actions and perceptions in the face of the COVID-19 threat but also affect the nationwide spread of COVID-19.

Note that the discrepancy of COVID-19 severity between Republican- and Democratic-leaning states was not overall significant during the post-Omicron period. Besides, the severity of COVID-19 risk in Democratic-leaning states was higher than that in Republican-leaning states, which is different from the case before the Omicron predominated. Some relevant reasons could be as follows. 1) Relaxation of requirements for mask-wearing and social distancing. Earlier in May 2021, the CDC said that people who were fully vaccinated didn't need to wear a mask or kept a distance of 6 feet from others [34]. When the Omicron variant appeared, President Joe Biden told people that it was a cause for concern rather than panic [35]. Later in February 2022, the CDC further eased COVID-19 guidelines for masks, which meant that 72% of the population lived in communities no longer recommended indoor masks [36]. Such guidelines and propaganda could be seriously followed by Democratic-leaning people and cause plenty of extra-confirmed cases, based on the findings of Kahane [33] and Young et al. [32] among others. 2) Deficiency of vaccine protection. Vaccines most people get were supposed to work on pathogens such as Delta before Omicron appeared. However, recent studies found that these types of vaccines own less protection for the Omicron variant [37] unless the third dose or a first booster was vaccinated [38, 39]. Data from WHO [16] showed that as of October 11, 2022, only 32.47% of persons have received a first booster dose or the third dose. Therefore, in the first wave of Omicron, combined with the first possible reason, the number of confirmed cases in Democratic-leaning states would increase more rapidly than before. 3) Change of perceptions for the risk of COVID-19. Increasing literature revealed that the severity of suffering from Omicron was much milder than other pathogens like Delta no matter in children [40] adults [41] or elder patients [42]. In light of the fact that previous waves of COVID-19 had caused great economic impacts [43, 44], perceptions of the threat of COVID-19 would have inevitably changed, which could also reduce the willingness to test COVID-19. In this regard, combing with the previous reasons, the number of confirmed cases could further increase.

Some notes of this present research should be highlighted. First, the analysis process is carried out based on state-level cases and it could be better to use county-level data to get more informative messages. Second, since the data analyzed here heavily relies on the quality of surveillance data–-the cumulative confirmed cases, the outcomes could be biased due to the unreported cases [45]. Third, it is easy to find that compared with Republican-leaning states, Democratic-leaning states have overall obviously different severity of the COVID-19 threat before and after the emergence of Omicron and the possible reasons should be further investigated.

Even if the present research is not flawless, some findings are still meaningful for both masses and policymakers. One can realize the risk of COVID-19 is still high in both Democratic- and Republican-leaning states. Therefore, individuals still should take some actions such as wearing a high-quality mask to minimize the possibility of infection even if the CDC has dropped quarantine and social distancing recommendations [46]. Since phenomena like pseudoscience, conspiracy theories, and skepticism of vaccination policies are often correlated to conservatives, it is meaningful for governments to disseminate the effect of vaccination with less promotional information that could make conservatives less supportive [47] and simultaneously crack down on persons who spread false information to correct the misperception of the COVID-19 threat.

## Data Availability

State-level COVID-19 data: https://data.cdc.gov/Case-Surveillance/United-States-COVID-19-Cases-and-Deaths-by-State-o/9mfq-cb36/data; MIT election data: https://dataverse.harvard.edu/dataset.xhtml?persistentId=doi:10.7910/DVN/42MVDX; State-level population data: https://www.census.gov/data/tables/time-series/demo/popest/2020s-state-total.html
